# Directional Scattering of Semiconductor Nanoparticles Embedded in a Liquid Crystal

**DOI:** 10.3390/ma7042784

**Published:** 2014-04-03

**Authors:** Braulio García-Cámara, José Francisco Algorri, Virginia Urruchi, José Manuel Sánchez-Pena

**Affiliations:** Displays and Photonic Applications Group, Department of Electronic Technology, Carlos III University of Madrid, Avenida de la Universidad 30, Leganés E28911, Spain; E-Mails: jalgorri@ing.uc3m.es (J.F.A.); vurruchi@ing.uc3m.es (V.U.); jmpena@ing.uc3m.es (J.M.S.-P.)

**Keywords:** semiconductor nanoparticles, liquid crystals, directional scattering, Mie resonances

## Abstract

Light scattering by semiconductor nanoparticles has been shown to be more complex than was believed until now. Both electric and magnetic responses emerge in the visible range. In addition, directional effects on light scattering of these nanoparticles were recently obtained. In particular, zero backward and minimum-forward scattering are observed. These phenomena are very interesting for several applications such as, for instance, optical switches or modulators. The strong dependence of these phenomena on the properties of both the particle and the surrounding medium can be used to tune them. The electrical control on the optical properties of liquid crystals could be used to control the directional effects of embedded semiconductor nanoparticles. In this work, we theoretically analyze the effects on the directional distribution of light scattering by these particles when the refractive index of a surrounded liquid crystal changes from the ordinary to the extraordinary configuration. Several semiconductor materials and liquid crystals are studied in order to optimize the contrast between the two states.

## 1. Introduction

Active plasmonics has recently appears as an emergent research field [1]. The active tuning of plasmonic phenomena could be determinant for the design and development of all-optical devices for manipulating light at the nanoscale. Plasmon resonances could be modified through the variation of the size, shape or composition of the nanoparticle or by changing the refractive index of the surrounding medium. While any change in the geometrical and structural properties of the particle introduces static modifications, the variation of the effective refractive index of the host medium can be dynamic, reversible, and controllable [2]. Liquid crystals are probably one of the most important active dielectric media, due to their small elastic constant and high birefringence [3], thus their refractive index can be easily modified. In addition, there are several ways to control it. Electrically-controlled [4], light-controlled [5] and also thermal-controlled [6] techniques have been reported to modify both the refractive index of a liquid crystal and then the plasmon resonance of nanoparticles embedded on it. This has led to the emergence of active devices based on complex metamaterials composed of plasmonic nanostructures embedded in LC [7]. For instance, optical switches [8,9], modulators [10,11] or filters [12], among others.

Recent works have shown that semiconductor nanoparticles, e.g., silicon, germanium, *etc.*, present Mie resonances in light scattering with a similar behavior to plasmon polaritons [13,14]. The interest on these phenomena has grown rapidly due to important advantages respect with plasmonic nanoparticles. This first one is that semiconductor nanoparticles has lower absorption in the visible range than metallic ones; thus, thermal effects on them are less important. Although these effects are interesting for some applications such as photothermal therapies, the heating suffering the nanostructures produce degradation. The second one is the CMOS compatibility of semiconductors and then, the easy integration of this kind of devices on chips. In addition, these nanoparticles have a magnetodielectric response in the visible range, never observed before. They present both electric and magnetic response in this range [13]. This effect has certainly been a wake-up call in the nanoscience field. Coherent effects between electric and magnetic contribution, producing certain control over the spatial distribution of the scattered light, were suggested more than 30 years ago [15]. However, they were unable to be experimentally demonstrated until now. Both zero-backward and minimum-forward scattering has been finally observed in the laboratory [16–18]. The control of directionality of light scattering can be very useful for applications ranging from biosensors [19] to all-optical devices for computing [20,21] or renewable energies [22].

Herein, we numerically study the possibility to control the directionality of light scattering of semiconductor nanoparticles embedded in a liquid crystal through controlling the optical anisotropy of this active medium via an external electric field. For this task, we have considered semiconductor nanoparticles for which the directionality conditions proposed by Kerker and coworkers [15] are satisfied in the visible range. In order to maximize the change in the scattered energy at certain directions, several materials and liquid crystals have been considered.

## 2. Theoretical Background

The electromagnetic scattering by a homogeneous and isotropic sphere of radius *r* illuminated by a linearly polarized plane wave of wavelength λ is properly explained in the Lorentz-Mie theory [23,24]. The different dependences of light scattering on the properties of both the particle and the surrounding medium are quite interesting for a great range of applications. These dependences are usually studied through the extinction and scattering efficiencies. These parameters can be expressed as a multipolar expansion given by [24]:

$$\begin{array}{l} Q_{sca}=\frac{2}{x^{2}}\sum_{n=1}^{\infty }(2n+1)\left({\left|a_{n}\right|}^{2}+{\left|b_{n}\right|}^{2}\right)\\ Q_{ext}=\frac{2}{x^{2}}\sum_{n=1}^{\infty }\left(2n+1\right)ℜ(a_{n}+b_{n}) \end{array}$$ (1)

where *a_n_* and *b_n_* are, respectively, the electric and magnetic n-polar Mie coefficients; and *x* is the size parameters, defined as *x = k*·*r; k* being the light wavenumber. Extinction refers to the part of the incident beam that is extinguished due to the two main light-matter interaction phenomena: scattering and absorption. Unlike metals, considered semiconductors present dominant scattering in the visible range. Thus, scattering and extinction are mainly equal in the present case. However, hereinafter, we will still consider the extinction efficiency as a representative parameter of light-matter interaction.

In addition, the spatial distribution of the scattered energy by the particle is also quite interesting due to its use as antennas [25]. The angular dependence of this energy is usually described via the differential scattering efficiency (*Q_diff_*) [24]:

$$Q_{diff}=\frac{1}{x^{2}}\left\{{\left|\sum_{n}\frac{\left(2n+1\right)}{n(n+1)}(a_{n}\pi_{n}+b_{n}\tau_{n})\right|}^{2}+{\left|\sum_{n}\frac{\left(2n+1\right)}{n(n+1)}(a_{n}\tau_{n}+b_{n}\pi_{n})\right|}^{2}\right\}$$ (2)

where π*_n_* and τ*_n_* introduce the angular dependence through the Legendre functions of first kind. The particular cases of the differential scattering efficiencies at the forward and backward directions are described via the forward scattering (*Q_FS_*) and the radar backscattering (*Q_RBS_*) efficiencies, and are given by [20]:

$$\begin{array}{l} Q_{RBS}=\frac{1}{x^{2}}{\left|\sum_{n}\left(2n+1\right){\left(-1\right)}^{n}(a_{n}-b_{n})\right|}^{2}\\ Q_{FS}=\frac{1}{x^{2}}{\left|\sum_{n}\left(2n+1\right)(a_{n}+b_{n})\right|}^{2} \end{array}$$ (3)

For subwavelength particles, this is nanoparticles in the visible range; the multipolar expansion can be well approximated by the first two electric and magnetic terms (*a*_1_, *a*_2_, *b*_1_ and *b*_2_). Under this approximation, previous expression can be reduced in the following way. The extinction efficiency of the nanoparticle is given by:

$$Q_{ext}=\frac{2}{x^{2}}[3\text{Re}(a_{1}+b_{1})+5\text{Re}(a_{2}+b_{2})]$$ (4)

whereas the *Q_diff_* is described by

Qdiff=1x2{|32(a1+b1cosθ)+56(3a2cosθ+6b2cos2θ−3b2)|2 +|32(a1cosθ+b1)+56(6a2cos2θ−3a2+3b2cosθ)|2} (5)

Finally, the *Q_RBS_* and *Q_FS_* for such small particle have the following expressions [24]:

$$\begin{array}{l} Q_{RBS}=\frac{1}{x^{2}}{\left|-3(a_{1}-b_{1})+5(a_{2}-b_{2})\right|}^{2}\\ Q_{FS}=\frac{1}{x^{2}}{\left|3(a_{1}+b_{1})+5(a_{2}+b_{2})\right|}^{2} \end{array}$$ (6)

Coherent effects can occur between different dipolar and quadrupolar terms in the forward and backward directions. In particular, previous works showed that the electric and the magnetic dipoles can interfere constructively or destructively producing minima in either the backward or the forward scattered intensity at certain light frequencies [15]. The relationships between the first two Mie coefficients to achieve the zero-backward or a minimum forward scattering are denoted as generalized Kerker’s conditions. These are easily predicted from Equation (6) and are described as [15,26]:

$$\begin{array}{c} a_{1}=b_{1}\\ ℜ(a_{1})=ℜ(b_{1})\;and\;ℑ(a_{1})=-ℑ(b_{1}) \end{array}$$ (7)

These conditions were unachievable for several years, because conventional materials did not present both electric and magnetic response in the visible range. However, very recently these phenomena are observed in semiconductor particles [16–18].

On the other hand, nematic LC is a complex medium composed of long helical rods or organic molecules producing a certain order in the direction of the components, labeled by a unit vector, or the director *n*. This anisotropic structure involves a uniaxial optical symmetry with two principal refractive indexes, the ordinary refractive index (*n_o_*) and the extraordinary refractive index (*n_e_*). While *n_o_* it is seen for electromagnetic radiation with the electric field perpendicular to the director, *n_e_* is related to polarized radiation with the electric field parallel to the director [3]. This optical anisotropy is usually characterized through the birefringence parameter, which is defined as Δ*n* = *n_e_* *− n_o_*. The variation of the refractive index seeing by an incident beam can be controlled by the polarization of light, but also by electric-field driven methods [2,4] or heat-driven methods [2,6], among others. In particular, we have considered the nematic LC E7 that is widely used. This is a mixture composed of 4-cyano-4’-n-pentyl-biphenyl (5CB), 4-cyano-4’-nheptyl-biphenyl (7CB), 4-cyano-4’-n-octyloxy-biphenyl and 4-cyano-4’’-n-pentyl-p-terphenyl. It has a birefringence of 0.2 in the visible range and works in the nematic phase in a wide temperature range, from −10 to 59°C [27]. In addition, its dielectric anisotropy is positive (Δε = 13.8) and it is commercially available [4].

## 3. Results and Discussion

**Figure 1.**
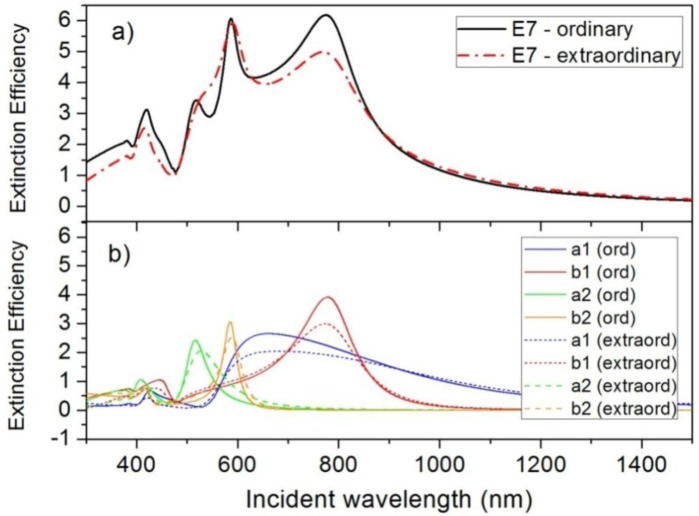
(**a**) Extinction efficiency of a silicon nanoparticle (*R* = 100 nm) embedded in an E7 LC when either *n_e_* or *n_o_* is considered; (**b**) four first multipolar contributions of the extinction efficiency when either the ordinary or extraordinary state of E7 LC is considered.

Light scattering by high-refractive-index semiconductor nanoparticles (e.g., silicon, germanium) with radius of few hundreds of nanometers presents Mie resonances for incident wavelengths in the visible range, as was commented above. In contrast with plasmonic nanoparticles, the spectrum of the scattered radiation by semiconductor nanoparticles is more complex, showing both electric and magnetic resonances [13,14]. However, the spectral behavior is similar and they depend on both the size and shape of the nanoparticles, and the refractive index of the surrounding medium. This allows the use of active mediums, such as liquid crystals, to tune the scattering properties of semiconductor nanoparticles. Figure 1a shows the extinction efficiency, *Q_ext_*, of a silicon nanoparticle with a radius of *R* = 100 nm embedded in a nematic LC, in particular, in an E7 LC. Both ordinary and extraordinary states of the LC are considered. Experimental refractive indexes have been used for these and other semiconductor materials and liquid crystals. They were obtained from references [28,29], respectively. As can be seen, the shift from the ordinary to the extraordinary state, meaning a change of the refractive index of the LC from 1.52 to 1.73 at room temperature [29], produces a slight modification of the extinction spectrum. In order to obtain a deep knowledge of the variations as well as the origin of each resonant peak, the spectral evolution of the first four dipolar contributions of the extinction efficiency (see Equation (4)) are plotted in Figure 1b. The magnetic modes, related to Mie coefficients *b*_1_ and *b*_2_ are predominant and the change from the ordinary to the extraordinary state only produces on them a small decrease of the maximum value. Any spectral shift is negligible. Electric modes, related to *a*_1_ and *a*_2_, suffer both a slight spectral shift and a decrement of the maximum *Q_ext_*. As was commented above, these resonances are mainly presented in light scattering, unlike metals whose resonances are mainly related to the absorption.

**Figure 2.**
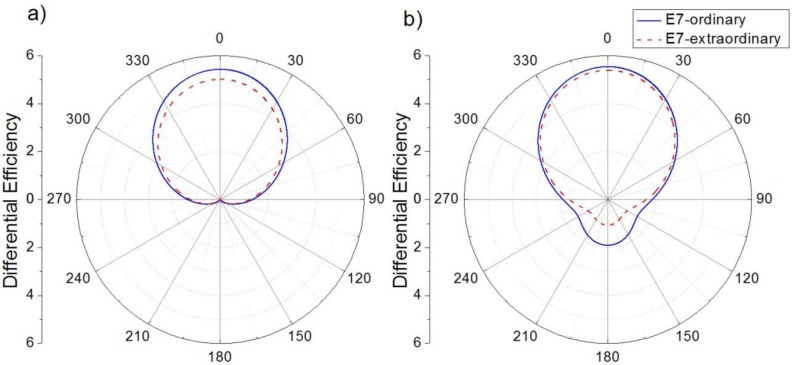
Differential efficiency (*Q_diff_*) of a single silicon nanoparticle (*R* = 100 nm) embedded in an E7 nematic LC when the incident wavelength satisfies (**a**) the first Kerker’s condition and (**b**) the second Kerker’s condition. The ordinary (solid blue lines) and the extraordinary (dashed red line) index of E7 LC are considered.

The existence of both electric and magnetic resonances in light scattering of semiconductor nanoparticles allows the appearance of coherent effects. As was previously cited, interferential phenomena between the dipolar electric and magnetic contributions produce anisotropy in the angular distribution of the scattered radiation. In particular, almost null backward and minimum forward scattering can be obtained when the dipolar Mie coefficients satisfy the expressions of Equation (7), respectively [15,26]. From Figure 1b, it seems that these conditions could be fulfilled at these wavelengths for which *a*_1_ and *b*_1_ contributions cross each other. Although the changes in the *Q_ext_* spectrum, when the surrounding refractive index changes from *n_o_* to *n_e_*, are not remarkable, the slight shifts could affect the satisfaction of these conditions. Checking the scattering patterns at these wavelengths, we can observe that Kerker’s conditions are satisfied at 840.9 nm and 719.7 nm, respectively, when the incident light sees the ordinary refractive index of E7 LC. If the state of the LC is modified such that the incident light sees its extraordinary index, wavelengths of Kerker’s conditions shift to 840.8 nm and 700.7 nm, respectively. As can be seen, while the zero-backward condition is quite steady, the minimum-forward condition is much more dependent on the boundary conditions [30]. Figure 2 attempts to show how these variations affect the angular distribution of the scattered light. Figure 2a shows *Q_diff_* of the silicon nanoparticles at λ = 840.9 nm when the surrounding refractive index corresponds with either *n_o_* (solid blue line) or *n_e_* (dashed red line) of E7 LC. Figure 2b shows *Q_diff_* when the incident wave fulfills the second Kerker’s condition, λ = 719.7 nm, under both refractive indices of E7 LC. While the zero-backward condition (Figure 2a) is almost unalterable with a deep decrease of light scattering in the backward direction (θ = 180°) at both considered scenarios, the distribution of the scattered radiation under the minimum-forward condition (Figure 2b) slightly changes, in particular in the backward hemisphere (*θ* = 90°–270°). As it was related in several works [16,30], the forward condition is hard to obtain. Moreover, its observance strongly depends on the refractive index of the surrounding medium [19]. For this reason, as the refractive index of E7 LC ranges from 1.5 to 1.7, a proper minimum-forward scattering cannot be observed.

**Figure 3.**
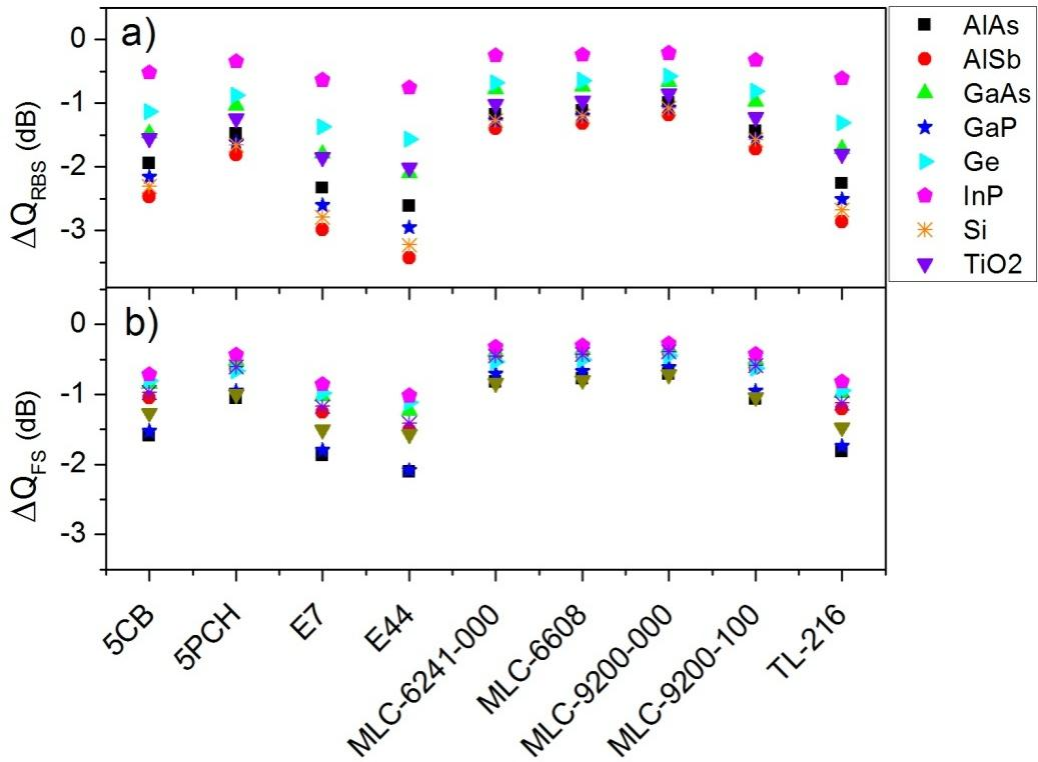
Variation of (**a**) the radar backscattering efficiency (*Q_RBS_*) and (**b**) the forward scattering efficiency, in dB, of a spherical particle of *R* = 100 nm embedded in a LC that changes its refractive index from the ordinary to the extraordinary value *versus* the surrounding LC. The incident wavelength satisfies the first and second Kerker’s conditions, respectively, when the LC presents the *n_o_*. Several semiconductor materials are considered.

Previous polar plots do not show remarkable changes in the scattered radiation at the forward and backward directions when the state of the LC changes. Similar results are obtained for other semiconductor nanoparticles embedded in different LC. However, a more precise observance of these phenomena can be made through the analysis of the forward scattering (*Q_FS_*) and the radar backscattering (*Q_RBS_*) efficiencies. These parameters give us information about light scattering at these certain directions, and they can be expressed as a function of Mie theory, as can be seen in Equations (3) and (6) for a complete or approximated situation, respectively. As we are interested in the modification of these parameters when the refractive index of the LC changes by means of an electric control, variations of both efficiencies from the ordinary to the extraordinary configuration are considered. Figure 3a shows the variation of *Q_RBS_* of a particle with a radius of 100 nm and made of different semiconductors, expressed in dB, and as a function of the surrounding LC when the refractive index of the LC changes from the ordinary to the extraordinary one. The incident wavelength is such that the first Kerker’s condition is satisfied in the ordinary state. On the other hand, Figure 3b represents the change of *Q_FS_* when the incident wavelength satisfies the second Kerker’s condition in the ordinary state. As can be seen, larger variations are obtained in the backward scattering (Figure 3a). In particular, *AlSb* nanoparticles seem optimum for these tasks, presenting a maximum variation of −3.5 dB when they are embedded in *E44* LC. However, silicon nanoparticles also present large variations. Among the considered liquid crystals, *E7* and *E44* produce the maximum variations.

**Figure 4.**
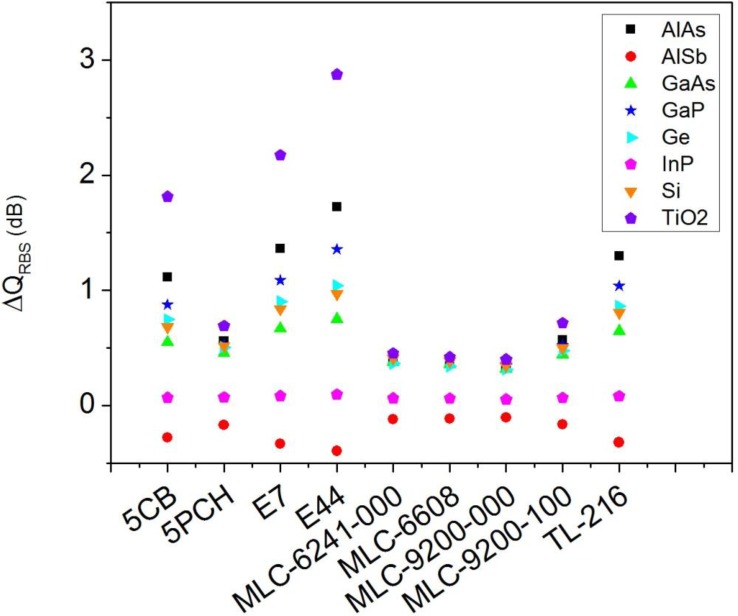
Variation of the radar backscattering efficiency (*Q_RBS_*), in dB, of a spherical particle of *R* = 100 nm embedded in a LC that changes its refractive index from the ordinary to the extraordinary value *versus* the surrounding LC. The incident wavelength satisfies the minimum-forward scattering condition when the LC presents the *n_o_*. Several semiconductor materials are considered.

From Figure 2b it can be seen that the larger variation of light scattering when the minimum-forward scattering condition is fulfilled does not occur in the forward direction (θ = 0°), if not in the backward direction (θ = 180°). For this reason, Figure 4 shows the variation of the *Q_RBS_* when the incident wavelength satisfies the second Kerker’s condition. In this case, the variation is positive and the differences between different LC and semiconductor materials are larger. In this sense, TiO_2_ nanoparticles present maximum values when they are embedded in 5CH, E7 or E44.

## 4. Conclusions

Active plasmonic devices have arisen for several applications. These devices usually considered plasmon structures, e.g., metallic nanoparticles or structures, embedded in electrically and optically active media, for instance liquid crystals. The recent discover of resonant behaviors in light scattering of semiconductor nanospheres have been appeared as an important stimulus for Plasmonics. In particular, the presence of both electric and magnetic allows the appearance of coherent effects and thus a certain control over the spatial distribution of the scattered energy by these particles. In this work, we have analyzed the possibility to vary these directional behaviors of light scattering by embedding these semiconductor nanoparticles in a LC. We have observed that the influence of the refractive index of the LC when it changes from its ordinary to the extraordinary value is weak for the considered LC. Maximum values of −3.5 dB are observed. In addition, the relative high refractive index of LC frustrates a minimum-forward scattering compared with other scattering directions. For these reasons, the analysis of backward scattering is more appropriate. Present results could be optimized through the use of high-birefringence and low-refractive-index liquid crystals. The electric control over the spatial distribution of light scattering could be useful for several applications, e.g., optical switches; therefore, its analysis is interesting.
